# Molecular analysis and antimicrobial resistance pattern of distinct strains of *Pseudomonas aeruginosa* isolated from cystic fibrosis patients in Iran

**Published:** 2019-04

**Authors:** Mohammad Emaneini, Davood Kalantar-Neyestanaki, Leila Jabalameli, Mojtaba Hashemi, Reza Beigverdi, Fereshteh Jabalameli

**Affiliations:** 1 Department of Microbiology, School of Medicine, Tehran University of Medical Sciences, Tehran, Iran; 2 Department of Microbiology, School of Medicine, Kerman University of Medical Sciences, Kerman, Iran; 3 Department of Microbiology, Karaj Branch, Islamic Azad University, Karaj, Iran; 4 Department of Pediatric Gastroenterology, School of Medicine, Arak University of Medical Sciences, Arak, Iran

**Keywords:** *Pseudomonas aeruginosa*, Type 3 secretion system, Cystic fibrosis, Cytotoxicity, Biofilm

## Abstract

**Background and Objectives:**

Colonization of *Pseudomonas aeruginosa* in Cystic Fibrosis (CF) patients may lead to severe pulmonary disease and death. Different characteristics of *P. aeruginosa* from these patients were determined in the present study.

**Materials and Methods:**

Antimicrobial susceptibility and AmpC-overproduction were determined. The β-lactamase genes were detected by PCR and the *oprD* gene was sequenced in some of the carbapenem resistance isolates. Distribution of *exo* genes was determined by PCR. Cytotoxicity of Exo effector proteins was measured using A549 cells. Biofilm production was determined by microtiter plate assay. Random amplified polymorphic DNA (RAPD) –PCR was performed for molecular analysis.

**Results:**

Polymyxin B, piperacillin/tazobactam and meropenem were the most active antibiotics and 9.6% of isolates were *ampC* overproducers. The prevalence of *bla*_VEB_, *bla*_OXA_, *bla*_VIM_, and *bla*_PER_ genes were as follow: 22.7%, 3.75%, 6.25% and 3.75%, respectively. A high proportion (83.5%) of isolates was able to produce biofilm. The *exoT* gene was present in all isolates while *exoU* was present in about 35% of them. RAPD-PCR revealed 49 patterns among 78 tested isolates in which 34 patterns were detected once.

**Conclusion:**

Biofilm formation ability and relatively high frequency of *exoS* may contribute to the persistence of bacteria within lungs of CF patients. Some characteristics of isolates recovered from a single patient after several sampling procedures were similar, while others lacked resemblance.

## INTRODUCTION

*Pseudomonas aeruginosa*, a major opportunistic pathogen causing a diverse range of human infections, is the most frequently isolated pathogen from sputum of patients with cystic fibrosis (CF) lung disease (1). Among multiple pathogenic elements involved in infection process (2), type III secretion system (T3SS) effectors have been well studied and recognized as key for establishing infection (3). Currently, four type III-secreted effector proteins, ExoS, ExoT, ExoU, ExoY, have been recognized in *P. aeruginosa* (4, 5, 6). Both ExoT and ExoS show ADP-ribosyltransferase and GTPase activating activities. They result in inhibition of actin polymerization, prevent phagocytosis and cell migration, and promote apoptosis (7). ExoU and ExoY have phospholipase and adenylate cyclase activities, respectively (8, 9). Together, these secreted toxins result in disruption of key eukaryotic cellular signaling pathways (10). Furthermore, in clinical studies, the presence of these exoenzymes is associated with a dissatisfactory clinical outcome among patients with *P. aeruginosa* infection (9). A great deal of evidence has demonstrated that the biofilm formation is responsible, in part, for therapeutic failure and biofilms have been shown to be up to 1000 times more resistant to antibiotics than planktonic, or free-swimming, cells of the same isolate (11). Presence of quorum-sensing molecules in CF sputum, used by *P. aeruginosa* and other bacteria for cell to cell communication *in vitro* (12), has been cited as evidence that *P. aeruginosa* in the CF lung form biofilms (13). On the other hand, due to increased multi-drug resistant isolates and biofilm formation capacity, treatment of infections caused by this bacterium is difficult, so therapeutic options are limited and a careful selection of antibiotic is necessary (14). β-lactam antibiotics are used for the treatment of acute pulmonary exacerbations in CF patients (15). Resistance mechanisms of *P. aeruginosa* to β-lactam antibiotics are multifactorial, including production of β-lactamase, AmpC over-production, loss of OprD porin and overexpression of efflux-pumps (16).

The distribution of T3SS effector genes amongst clinical isolates of *P. aeruginosa* remains to be elucidated. Furthermore, the prevalence of effector genes in populations of isolates from different disease sites has not been systematically and thoroughly explored. Hence, in this report, we investigate the distribution of the type III effector proteins-encoding genes and association between cytotoxicity and *exo* genes in *P. aeruginosa* strains obtained from cystic fibrosis patients. Other characteristics of isolates such as antibiotic resistant pattern as well as mechanisms of resistance to β-lactam antibiotics, biofilm capacity, and RAPD analysis were also determined.

## MATERIALS AND METHODS

### Bacterial isolates.

A total of 85 clinical isolates of *P. aeruginosa* were included in the present study. These isolates were collected from 27 CF patients attending the Children's Medical Center Hospital in Tehran, among which, six patients took part in collection process more than once. The collection process was carried out through April 2010 to June 2011. Primary identification to the species level were done based on colony morphology, pigment production, and Gram staining. All phenotypically different isolates were collected to be observed, though some of them were obtained from one patient. Routine bio-chemical tests used for confirmation were as follow: the ability of oxidase and catalase production, oxidative-fermentative (OF) test, and growth at 42°C on Mueller–Hinton agar (17).

### Antibiotic susceptibility test.

The antimicrobial susceptibility profile of isolated *P. aeruginoase* was determined by using antibiotic containing disks on Mueller Hinton agar according to Clinical and Laboratory Standards Institute (CLSI) guidelines (18). The disks contained the following antibiotics: ceftazidime (30 μg), aztreonam (30 μg), imipenem (10 μg), meropenem (10 μg), amikacin (30 μg), gentamicin (10 μg), tobramycin (10 μg), piperacillin/tazobactam (100/10 μg), ciprofloxacin (5 μg), levofloxacin (5 μg), ticarcillin (75 μg), polymyxin B (300 Unit). Determining of Multidrug resistance (MDR) isolates, as an important and problematic concern, were done based on considering resistance against at least 1 agent in ≥ 3 antimicrobial categories (19).

### Detection of *bla* genes among β-lactam resistant isolates.

Genomic DNAs used as template were extracted using DNA Extraction Kit (Accu-Prep® Genomic DNA Extraction Kit, USA Bioneer, Co.) according to the manufacturer's instructions. The *bla*_PER_, *bla*_SHV_, *bla*_VEB_, *bla*_PSE_, *bla*_CTX-M_, *bla*_OXA_, *bla*_OXA-48_, *bla*_VIM_, *bla*_IMP_, *bla*_GIM_, *bla*_AIM_, *bla*_SPM_, *bla*_NDM_, *bla*_SIM_, *bla*_KPC_ and *bla*_GES_ genes were detected by PCR. Amplification of β-lactamases genes was performed using specific primers (16, 20). The amplified PCR products were subjected to electrophoresis in 1.2% agarose gels, stained with ethidium bromide, and visualized in a Gel Doc XR molecular imager (Bio-Rad Laboratories).

### Detection of AmpC overproducer among carbapenem resistant isolates.

AmpC overproduction among carbapenem resistant isolates was assayed according to Rodríguez-Martínez et al. method (21). Briefly, the isolates were considered as AmpC over-producers when there was at least a two-fold dilution difference between the MICs (minimal inhibitory concentrations) of IMI (imipenem), MEM meropenem) or CAZ (ceftazidime) and MICs of these antibiotics plus 250 μg COL (cloxacillin).

### Sequencing of *oprD* gene among carbapenem resistant isolates.

PCR amplification of *oprD* among carbapenem resistant isolates was performed using specific primers (21). Sequences were compared with that of reference *P. aeruginosa* PAO1.

### Detection of genes encoding type-III secretion toxins.

Sequences of specific primers used in amplifying genes encoding type-III secretion toxins, *exoT*, *exoU*, *exoS*, *exoY*, based on PCR technique as previously described (22, 23).

### Cytotoxicity assay.

To determine cytotoxic effect of *P. aeruginosa* isolates on A549 human lung epithelial cells, isolated bacteria were grown in Luria-Bertani broth. Cultures were centrifuged and resultant pellets were washed three times with sterile PBS. The final bacterial cell pellets were re-suspended in PBS to yield the final concentration of 1.5 × 10^8^ cfu/ml. Human cells suspension were seeded on 96-well flat-bottom tissue culture plates at a density of 1.5 × 10^5^ cells / well with DMEM (Dulbecco's Modified Eagle's medium) and 10% Fetal Bovine Serum (FBS). After an overnight incubation of cell culture at 37°C, wells were rinsed with sterile PBS, followed by the addition of DMEM without phenol red and 1% FBS. Consequently, the diluted bacterial suspension of each *P. aeruginoasa* isolates were added to the cells and incubated at 37°C for 4 h. PBS without inoculated bacteria was used for negative controls. Eventually, lactate dehydrogenase activity was assayed for quantification of cytotoxicity using the Cytotoxicity Detection Kit^plus^ (Roche, Germany) according to the manufacturer's instructions. The assay was performed in triplicate.

### Biofilm formation.

Biofilm formation ability of isolates was determined based on a colorimetric microtiter plates assay as described by Lee et al. (24). In brief, an overnight bacterial culture in Tripticase Soy Broth (TSB) at 37°C was diluted (1:100) by fresh TSB and then inoculated into the sterile flat-bottomed 96-well polystyrene microtiter plates. After an incubation period of 24h at 37°C without shaking, wells were gently washed three times with PBS. 99% methanol was used for biofilm fixation for 15 min, and then the plate was air-dried. Afterwards, Crystal violet 1% (CV) was added for biofilm staining for 20 min. In the next step, unbound CV was removed and the remaining was released by adding 33% acetic acid. Finally, the absorbance of solubilized CV was measured at 590nm using a microtiter plate reader. All the assays were tested in triplicate. Uninoculated medium was considered as control negative sample. The cut-off OD (ODc) was defined as three standard deviations above the mean OD of the negative control. In accordance with the results of microtiter plate tests, Biofilm producers were characterized as follows based on the optical density: non-biofilm producers (OD test <ODc), weak biofilm producers (ODc< OD < 2× ODc), moderate biofilm producers (2× ODc< OD < 4× ODc), and strong biofilm producers (4× ODc< OD).

### PCR-RAPD typing of isolates.

The RAPD-PCR technique was used as described by Bulkanov et al. (25). This fingerprinting was carried out by using arbitrary primers, RAPD-208 (5′-AGCGG-GCCAA-3′) and 272 (5′-ACGGCCGACC-3′) (16, 26, 27). This amplification was performed in T-100 Gradient (BioRAD) using PCR Master kit (AMPLIQON, Denmark) according to manufacture guideline, with the following cycling parameters: initial denaturation at 95°C for 5 min followed by 45 cycles of denaturation at 95°C for 1 min, annealing for 1 min at 38.5°C, extension at 72°C for 5 min. The final extension step was continued for another 2 min at 72°C. After visualization of amplified products, genotypic profile of each isolate was determined. BioNumerics software was used in pattern analysis and isolates with ≥85% similarities were considered as indistinguishable (one pattern) types and isolates with <85% similarities were taken as different types. RAPD types were designated numerically. Type-1 was the most frequent pattern among 49 detected patterns.

## RESULTS

Table 1 shows the antibiotic resistance patterns of *P. aeruginosa* isolates. As shown in Table 1, polymyxin B followed by piperacillin/tazobactam and meropenem were the most active antibiotic against the panel of isolates. Ceftazidime was effective on about 50% of the isolates. Less than half of the isolates were determined as MDR organisms.

**Table 1.** The antimicrobial susceptibility patterns of *P. aeruginosa* isolates

**Antibiotics**	**N (%) of isolates (n=85)**		

	**S**	**I**	**R**
Ceftazidime	29 (34.11)	12 (14.11)	44 (51.76)
Aztreonam	42 (49.41)	17 (20)	26 (30.59)
Imipenem	71 (83.52)	0	14 (16.47)
Meropenem	77 (90.58)	0	8 (9.41)
Amikacin	69 (81.17)	4 (4.7)	12 (14.12)
Gentamicin	53 (62.35)	2 (2.35)	30 (35.29)
Tobramycin	56 (65.88)	1 (1.17)	28 (32.94)
Piperacillin/Tazobactam	77 (90.58)	0	8 (9.41)
Ciprofloxacin	57 (67.05)	8 (9.41)	20 (23.5)
Levofloxacin	42 (49.41)	15 (17.64)	28 (32.9)
Ticarcillin	52 (61.17)	5 (5.88)	28 (32.94)
Polymyxin B	85 (100)	0	0

S, susceptible; I, intermediate; R, resistant

The prevalence of *bla*_VEB_, *bla*_OXA_, *bla*_VIM_, and *bla*_PER_ genes were as follow: 22.7%, 3.75%, 6.25%, and 3.75% respectively. All isolates were negative for *bla*_SHV_, *bla*_PSE_, *bla*_CTX-M_, *bla*_OXA-48_, *bla*_IMP_, *bla*_GIM_, *bla*_AIM_, *bla*_SPM_, *bla*_NDM_, *bla*_SIM_, *bla*_KPC_ and *bla*_GES_ genes. Coexistence of *bla*_VEB_ and *bla*_PER_ was observed in 2 isolates.

Also, among 14 carbapenem (imipenem and meropenem) resistant isolates, 5 (35.7%) were positive for *bla*_VIM_. In all carbapenem resistant isolates *oprD* was inactivated by mutations. 12 out of 14 carbapenem resistant isolates, mechanism of *oprD* inactivation resulted from 1–18 bp deletions and substitution mutations which leads to internal stop codon and frame-shift mutations and in two isolates *oprD* was not detected by PCR (Table 2). 8 out of 14 carbapenem resistant isolates (57.1%) were AmpC overproducers and showed decrease in MICs for ceftazidime, imipenem and meropenem in presence of cloxacillin (Table 2).

**Table 2.** Characteristics of carbapenem resistant isolates

**Isolates**	**MIC (μg/ml)**						**AmpC Overproduction**	***bla*_VIM_**	***oprD* Mutation**

	CAZ^*^	**CAZ-COL**	**IMI**	**IMI-COL**	**MEM**	**MEM-COL**			
P1-1	32	32	32	16	1	1	−	−	1.N.D^†^
P1-2	8	2	16	16	16	16	+	−	1.N.D
P1-3	8	8	16	16	16	16	−	+	6.N.D
P1-4	16	16	8	8	1	1	−	−	12.N.D
P1-7	256	256	256	256	128	128	−	−	Not-D^‡^
P1-8	2	2	16	8	8	8	−	+	18.N.D
P1-9	256	256	512	256	256	256	−	−	Not-D
P1-10	256	256	32	16	8	1	+	+	5.N.D
P3-2	32	32	32	16	8	1	+	−	4.N.D
P3-5	2	2	16	1	1	1	+	−	11.N.D
P14-1	32	16	32	8	8	1	+	+	1.N.D
P14-2	2	2	8	1	1	1	+	+	7.N.D
P17-9	2	2	8	1	1	1	+	−	1.N.D
P17-10	2	2	8	1	1	1	+	−	6.N.D

* CAZ, Ceftazidime; CAZ-COL, Ceftazidime-Cloxacillin; IMI, Imipenem; IMI-COL, Imipenem-Cloxacillin; MEM, Meropenem; MEM-COL, Meropenem - Cloxacillin

† Nucleotide Deletion,

‡ Not Determined

Different characteristics of *P. aeruginosa* isolates including type III secretion-toxin encoding gene patterns, biofilm formation and cytotoxicity effect are shown in Table 3. A high proportion of isolates were able to produce biofilm (83.5%) in which, 20%, 37.64% and 25.88% were determined as weak, moderate, and strong biofilm producers, respectively.

**Table 3.** Some characteristics of *P. aeruginosa* isolates

**Genes**				Biofilm density^*^	**% of cytotoxicity**			**N. of isolates**
	
***exoS***	***exoY***	***exoU***	***exoT***		**Range**	**50%^†^**	**90%**	
+	+	+	+	16 M	3.41–68.94	25.76	53.41	16
+	+	−	+	7 W, 16 M, 10 S, 6 N	0–81.06	17.63	39.39	39
−	+	+	+	5 S,3 W	0–81.06	16.167	24.62	8
−	−	−	+	8 W	0–21	14.77	15.91	8
−	−	+	+	4 W	36.74–81.06	37.88	50.74	4
−	+	−	+	2 W	24.38–22.54	-	-	2
+	−	+	+	2 S	23.11–39.77	-	-	2
+	−	-	+	5 S, 1 N	6.12–61.36	7.2	61.36	6

* W, weak; M, moderate; S, strong; N, negative

† The cytotoxicity 50: 50% of the isolates shows below this cytotoxicity value and the same principal for the cytotoxicity 90.

**Fig. 1.**
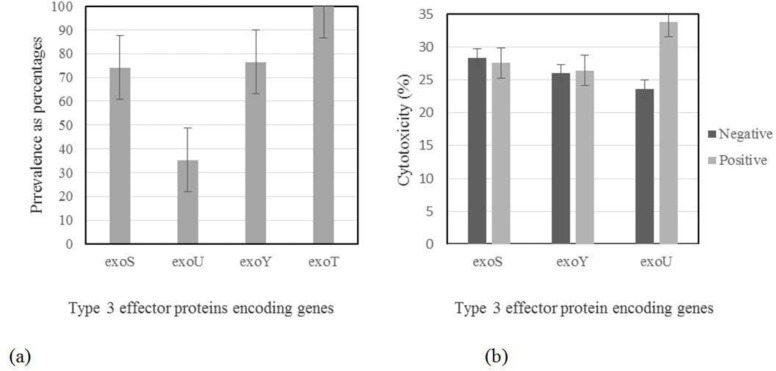
(a) Prevalence (as percentages) of the *exoS*, b: *exoU*, c: *exoY*, d: *exoT* genes among isolates. Error bars represent standard errors. (b) Correlation of *exo* genes existence with cytotoxicity of A549 cells. Error bars represent standard errors. Because of observing *exoT* in all isolates, data related to this gene is not presented in this figure.

As shown in Fig 1a, *exoT* was present in all isolates. Most isolates harbored *exoS* and *exoY*, while *exoU* was present in about 35% of isolates. *In vitro* cytotoxicity against A549 human lung epithelial cells was observed in strains carrying type III secretory genes. The correlation between the presence of *exo* genes and cytotoxicity is demonstrated in Fig. 1b. It revealed that levels of cytotoxicity were found to be significantly affected by presence of *exoU* gene.

**Fig. 2.**
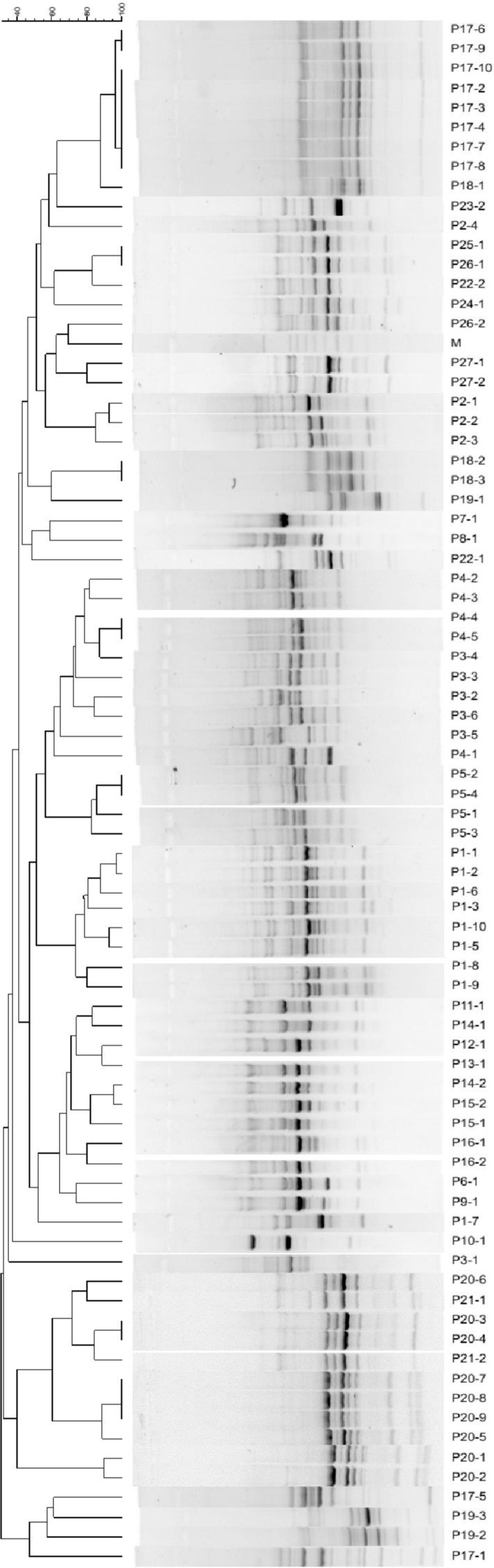
RAPD-PCR generated dendrogram for 78 *P. aeruginosa* isolates of 27 different patients.

Results based on RAPD-PCR, as a quick method to characterize the heterogeneity in the group by using a cut off value of 85% as the threshold, revealed 49 patterns among 78 tested isolates in which 34 patterns were detected once, while the remaining 15 patterns were repeatedly observed. RAPD-PCR generated dendrogram of 78 *P. aeruginosa* isolates from 27 different patients is illustrated in Fig. 2.

Properties of the isolates obtained from some of the patients who have been taking part in sampling for several times, during a year, are demonstrated in Table 4.

**Table 4.** Detailed properties of isolates obtained from patients who took part in samplings for several times

**Patients**	**Attending dates**	**Isolates**	Gene pattern of T3SS^*^	**Cytotoxicity (%)**	**Biofilm density^†^**	**Antibiotic resistant pattern^‡^**	**RAPD Type^§^**
P1	2010/7/6	P1-1	S+U^+^	15.2	-	ATM, IMI, GM, TM, CIP, LE, TC	8
		P1-2	S^−^U^+^	24.4	W	ATM, IMI, MEM, CIP, LE, TC	8
	2010/9/29	P1-3	S+U^−^	8.8	M	CAZ, ATM, IMI, MEM, GM, TM, LE, CPM	33
		P1-4	S+U^−^	7.2	W	CAZ, ATM, IMI, AK, GM, TM,TC	37
		P1-5	S+U^−^	7.0	W	CAZ, ATM, AK, GM, TM, TC	9
		P1-6	S+U^−^	17.9	M	CAZ, ATM, GM, TM	8
	2010/11/1	P1-7	S+U^−^	81.1	W	CAZ, ATM, IMI, MEM, GM, TM, CIP, TC	40
		P1-8	S+U^−^	11.8	M	ATM, IMI, MEM, GM, TM, CIP, TC	22
	2011/4/4	P1-9	S+U^+^	3.5	W	CAZ, ATM, IMI, MEM, AK, GM, TM, PTZ, CIP, TC	23
		P1-10	S+U^−^	73.2	W	CAZ, ATM, IMI, MEM, GM, TM, CIP, LE, TC	9
P14	2011/1/3	P14-1	S+U^−^	29.6	N	CAZ, IMI	12
	2011/3/21	P14-2	S+U^+^	28.5	W	ATM, IMI, MEM	12
P5	2010/7/29	P5-1	S+U^−^	23.2	W	CAZ, ATM, PTZ	7
	2010/9/17	P5-2	S+U^+^	14.1	W	CAZ	7
		P5-3	S^−^U^+^	81.1	M	CAZ, ATM, GM, TM, CIP, TC	51
	2010/10/25	P5-4	S+U^−^	20.9	S	CAZ	7
P3	2010/4/12	P3-1	S+U^+^	37.9	W	GM, LE	28
	2010/9/28	P3-2	S+U^−^	26.3	M	CAZ, IMI, MEM, LE	6
	2011/14/16	P3-5	S^−^U^+^	5.0	W	IMI, LE	6
		P3-6	S^−^U^+^	16.0	-	ATM, LE	50
P17	2010/7/20	P17-1	S^−^U^+^	37.9	-	CAZ, GM, TM, TC	38
		P17-2	S+U^−^	17.7	S	CAZ, GM, TM, TC	1
		P17-3	S+U^−^	15.6	S	CAZ, GM, TM, TC	1
		P17-4	S+U^−^	32.2	S	CAZ, GM, TM, TC	1
	2010/5/26	P17-5	S+U^−^	9.5	S	ATM, GM, TM, CIP, TC	44
	2010/10/5	P17-6	S+U^−^	-	M	GM, LE, TC	1
	2011/6/11	P17-8	S+U^−^	17.7	S	GM, TM, LE, TC	1
		P17-9	S+U^−^	11.8	S	ATM, IMI, GM, TM, CIP, TC	1
		P17-10	S+U^−^	10.3	S	ATM, IMI, GM, TM, CIP, LE, TC	1
P20	2010/7/9	P20-1	S^−^U^+^	22.7	M	LE	15
		P20-2	S+U^+^	57.2	S	CAZ, LE	15
		P20-3	S^−^U^+^	16.7	M	LE	13
	2010/9/8	P20-4	S+U^−^	39.4	S	CAZ, TC	13
		P20-5	S+U^+^	26.2	S	CAZ	14
		P20-6	S+U^−^	17.8	W	CAZ	35
	2011/4/19	P20-7	S^−^U^+^	16.7	M	CAZ	14
		P20-8	S^−^U^+^	50.8	S	CAZ	14
		P20-9	S^−^U^+^	24.62	W	CAZ, ATM, PTZ, LE, TC	14

* Type 3 secretion system,

† W, weak; M, moderate; S, strong; N, negative

‡ AK, Amikacin; ATM, Aztreonam; CTZ, Ceftazidime; CIP, Ciprofloxacin; GM, Gentamicin; IMI, Imipenem; LE, Levofloxacin; MEM, Meropenem; PTZ, Piperacillin/ Tazobactam; PB, Polymyxin B; TC, Ticarcillin; TM, Tobramycin.

§ Numerical types are obtained as a result of RAPD PCR method.

## DISCUSSION

As antibiotic resistant pattern shows, less than half of the present isolates were resistant to all tested antibiotics, except cefatazidime. The difference in antibiotic resistant pattern between the obtained results in the present and other studies (28) may be due to either the different common colones present in a certain region or different antibiotic treatment pattern. The frequency of MDR isolates in the present study was considerably less than the previous investigation done by our team on isolates from wound infections of burn patients (about 92%) (23). Results obtained by Rao et al. (28) even showed less frequency of MDR isolates from CF patients.

As shown in Table 2, AmpC overproduction and *oprD* mutations were associated with resistance to imipenem and meropenem. Furthermore, AmpC overproduction was associated with resistance to ceftazidime. In the present investigation, results revealed that the association between OprD gene deficiency and resistance to imipenem and meropenem is similar to other literatures. Mutations in *oprD* gene reportedly lead to increased MIC to imipenem (16).

To date, presence of different β-lactamases genes (TEM, SHV, CTXM, PER, VEB, GES and IBC families) were reported among *P. aeruginosa* isolates (16, 20). In the present study, VEB type of β-lactamases was the most predominant ESBLs. The results of this study demonstrated *bla*_VIM_ as the most common MBLs (Metallo-β-lactamases) found in carbapenem-resistant bacteria, including carbapenem-resistant *P. aeruginosa* in non CF patients (16, 20).

It was shown that type-III secretion system is an important virulence mechanism and cytotoxins encoded by *exoS, exoT, exoU* and *exoY* genes may be important contributors to the dissemination of the organism from the site of infection, bacterial evasion of the host immune response and inhibition of DNA synthesis leading to host cell death (29). The majority of *P. aeruginosa* strains carry *exoT* and *exoY* genes; however, the presence of *exoS* and *exoU* differ noticeably between the isolates and appear to be mutually exclusive (29). As the previous study done by our team on *P. aeruginosa* isolated from burn patients (23), *exoT* was the most prevalent cytotoxin gene found in isolates of CF patient and was reported in all isolates (100%), though *exoY* was not as frequent as *exoT* (about 79%). In contrast to our previous results that showed *exoS* as the least prevalent gene (29%) (23), present study revealed that *exoU* has less frequency than other cytotoxin genes (36.58%). Data showed that the least prevalence of *exoU* in CF patients, as a chronic disease, is in agreement with results reported by Feltman et al. (22), which may be due to the straight correlation between *exoU* and acute infections. So, the high prevalence of *exoU* gene in burn isolates, as was reported before is predictable. Relatively high frequency of isolates harboring *exoS* (about 80%) may be related to hypothesis presented by Feltman et al. (22). They suggested that production of *exoS* may provide *P. aeruginosa* isolates with an advantage in colonizing or persisting in CF lung. Furthermore, *exoS* may be genetically linked to a second factor that is important in the pathogenesis of *P. aeruginosa* pulmonary colonization and infection in CF patients. Taken together, our findings suggest that at least some of the genes encoding type III secretion effector proteins are present in all *P. aeruginosa* isolates, though the prevalence of them was different. Variation observed in the prevalence of *exo* genes may be the consequence of clinical specimen used for collecting isolates.

In the present study, results related to *in vitro* cytotoxic effect of strains carrying type III secretion proteins concur with the previous studies, in which the type III secretion system is reported to be the primary contributor to the virulence of *P. aeruginosa* and may lead to the increased morbidity and mortality in patients and animals (23).

High frequency of biofilm producers in the present study (more than 90%), as in other report, suggests that *P. aeruginosa* is able to form biofilms within the lungs of CF patients and this mode of growth likely facilitates persistence of the bacterium within this niche (30) and biofilm development in CF airway is one of the most striking features of *P. aeruginosa* adaptation to airways of patients with CF (31). However, CF isolates can be highly variable with respect to biofilm density. In the current study, the prevalence rate of strong biofilm producers was about 25%, whereas Perez et al. (32) could not find such a biofilm state among CF patients. No correlation was observed between biofilm formation and cytotoxicity.

Because of the presence of problems related to phenotypic variations of bacterial isolates throughout the period of infection in CF patients (33) and the difficulty of ascertaining the reinfection of patients or difficulty in determining whether the patient is infected by different strains over a period of time, or whether the isolated strains differ according to the respiratory tract site where the sample was taken (34), molecular typing was selected as a more sensitive and specific method in this study. RAPD typing of the 78 *P. aeruginosa* isolates gave 49 different RAPD fingerprints, 34 of which contained only 1 strain. The dominant RAPD type amongst the remaining 15 fingerprints contained 9 strains. Presence of most isolates harboring unique RAPD patterns may be due to the acquisition of these isolates from environmental sources where horizontal gene transfer occurs and may result in acquisition of new genes.

As shown in Table 4, our results revealed that more than one *P. aeruginosa* strains can be isolated from a CF patient in one time attendance for sampling, though a given strain was not recovered from a given patient after a period of time. In other words, isolation of different strains with different properties from one patient while attending for several times revealed the possibility of both phenotypic and genotypic alterations in causative strains. Furthermore, the similarity between some of the genotypic aspects of different strains, such as the same RAPD pattern in isolates 1 and 2 in patient 20 (P20) was observed, though another genotypic pattern related to *exo* genes was different. On the other hand, despite the presence of analogous *exo* genes and RAPD patterns in isolates P1-5 and P1-10, which were recovered from the same patient, their cytotoxic capability were drastically different. Moreover, recovery of an isolate with a similar RAPD pattern, 7, from patient 5, was observed in each round of sampling, though isolation of another different pattern, 51, from the mentioned patient, was restricted to a defined round of sampling. Interestingly, cytotoxicity of isolates with pattern 7 from patient 5, which were recovered constantly, was low and the last isolated strain with this pattern has a strong biofilm intensity. Failure of the treatment in CF patients may be due to the strong ability of causative *P. aeruginosa* to form biofilm. On the other hand, isolated strain with pattern 51, recovered once, from patient 5 was significantly more cytotoxic than other isolates with pattern 7. However, treatment and management of patient 5 was not acceptable and victorious. Such findings revealed that isolation of stable strains present in the whole duration of the disease along with new strains showing new different RAPD patterns in CF patients may be due to the acquisition of the latter strains from hospitals where they took part in sampling. Undoubtedly, antibiotic therapy against such new and transient strains may result in its elimination from patients, while stable strains with strong ability to form biofilm do not respond to such treatment and can be isolated for several times. However, it is well documented that in the initial stages of infection, when the bacteria are mainly planktonic and not yet persistent, their eradication may be easier and so it reveals the importance of early identification of infection (35).

## CONCLUSION

To our knowledge, the compelling role of biofilm formation capacity and cytotoxicity effect of isolates in bacterial invasion process validate probable and powerful therapeutic strategies other than conventional and traditional antibiotic treatment. Furthermore, relatively high frequency of antibiotic resistance among isolated *P. aeruginosa* may persuade the pharmaceutical industry to introduce new antibiotics to solve the resistance problem.
